# Dihydroxyphenylacetaldehyde Lowering Treatment Improves Locomotor and Neurochemical Abnormalities in the Rat Rotenone Model: Relevance to the Catecholaldehyde Hypothesis for the Pathogenesis of Parkinson’s Disease

**DOI:** 10.3390/ijms241512522

**Published:** 2023-08-07

**Authors:** Rawan Khashab, Naama Gutman-Sharabi, Zehava Shabtai, Regev Landau, Reut Halperin, Tsviya Fay-Karmon, Avshalom Leibowitz, Yehonatan Sharabi

**Affiliations:** 1 Hypertension Unit, Chaim Sheba Medical Center, Tel-HaShomer, Ramat Gan 5265601, Israel; rawan.khashab@sheba.health.gov.il (R.K.); n.sharabi3@gmail.com (N.G.-S.); zehava.shabtay@sheba.health.gov.il (Z.S.); regev.landau@gmail.com (R.L.); reut.halperin@sheba.health.gov.il (R.H.); tsviya.faykarmon@sheba.health.gov.il (T.F.-K.); avshalom.leibowitz@sheba.health.gov.il (A.L.); 2 Faculty of Medicine, Tel Aviv University, Tel Aviv 6997801, Israel

**Keywords:** Parkinson’s disease, DOPAL, catecholaldehydes, monoaminoxidase inhibitor, n-acetylcysteine

## Abstract

The catecholaldehyde hypothesis for the pathogenesis of Parkinson’s disease centers on accumulation of 3,4-dihydroxyphenylacetaldehyde (DOPAL) in dopaminergic neurons. To test the hypothesis, it is necessary to reduce DOPAL and assess if this improves locomotor abnormalities. Systemic administration of rotenone to rats reproduces the motor and central neurochemical abnormalities characterizing Parkinson’s disease. In this study, we used the monoamine oxidase inhibitor (MAOI) deprenyl to decrease DOPAL production, with or without the antioxidant N-acetylcysteine (NAC). Adult rats received subcutaneous vehicle, rotenone (2 mg/kg/day via a minipump), or rotenone with deprenyl (5 mg/kg/day i.p.) with or without oral NAC (1 mg/kg/day) for 28 days. Motor function tests included measures of open field activity and rearing. Striatal tissue was assayed for contents of dopamine, DOPAL, and other catechols. Compared to vehicle, rotenone reduced locomotor activity (distance, velocity and rearing); increased tissue DOPAL; and decreased dopamine concentrations and inhibited vesicular sequestration of cytoplasmic dopamine and enzymatic breakdown of cytoplasmic DOPAL by aldehyde dehydrogenase (ALDH), as indicated by DA/DOPAL and DOPAC/DOPAL ratios. The addition of deprenyl to rotenone improved all the locomotor indices, increased dopamine and decreased DOPAL contents, and corrected the rotenone-induced vesicular uptake and ALDH abnormalities. The beneficial effects were augmented when NAC was added to deprenyl. Rotenone evokes locomotor and striatal neurochemical abnormalities found in Parkinson’s disease, including DOPAL buildup. Administration of an MAOI attenuates these abnormalities, and NAC augments the beneficial effects. The results indicate a pathogenic role of DOPAL in the rotenone model and suggest that treatment with MAOI+NAC might be beneficial for Parkinson’s disease treatment.

## 1. Introduction

**Figure 1:**
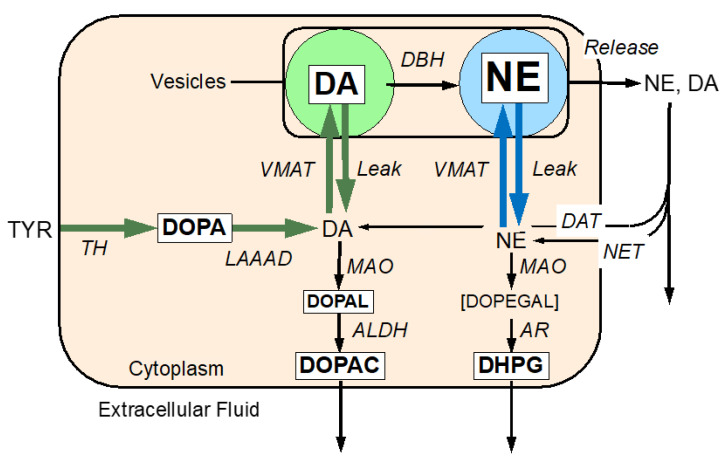
Concept diagram showing enzymatic steps in the synthesis, vesicular storage, release, reuptake, and metabolism of dopamine (DA) and norepinephrine (NE). DA is synthesized in the neuronal cytoplasmic via tyrosine hydroxylase (TH) acting on tyrosine to form DOPA and then L-aromatic-amino-acid decarboxylase (LAAAD) acting on DOPA. Most of cytoplasmic DA is taken up into vesicles via the vesicular monoamine transporter (VMAT), but a minority undergoes enzymatic oxidation catalyzed by monoamine oxidase (MAO) to form 3,4-dihydroxyphenylacetaldehyde (DOPAL). DOPAL is metabolized by aldehyde dehydrogenase (ALDH) to form 3,4-dihydroxyphenylacetic acid (DOPAC), which exits the cell. DA in the vesicles undergoes enzymatic hydroxylation catalyzed by DA-beta-hydroxylase (DBH) to form NE. Catecholamines released into the extracellular fluid is taken back up into the cytoplasm via the cell membrane DA transporter (DAT) or cell membrane NE transporter (NET). NE in the cytoplasm can undergo vesicular uptake or MAO-catalyzed oxidative deamination to form 3,4-dihydroxyphenylglycolaldehdyde (DOPEGAL), which is reduced by aldehyde/aldose reductase (AR) to form 3,4-dihydroxyphenylglycol (DHPG). DHPG rapidly exits the neuron. The six endogenous catechols in white rectangles were measured simultaneously. Font sizes correspond roughly to tissue concentrations of the analytes in rat striatum and peri-striatal tissue.

In Parkinson’s disease, it is generally accepted that nigrostriatal deficiency directly and solely reflects neuronal loss. The extent of catecholamine depletion in Parkinson’s disease, however, is greater than can be accounted for by denervation alone [1]. This discrepancy suggests that in the putamen there is a population of dysfunctional but extant residual dopaminergic neurons that are “sick but not dead”—and therefore potentially salvageable [1,2]. These abnormalities may be related to cytoplasmic buildup of the catecholaldehyde 3,4-dihydroxyphenylacetaldehyde (DOPAL). DOPAL, the immediate product of the action of monoamine oxidase (MAO) on cytoplasmic dopamine, is toxic to catecholaminergic neurons (Figure 1) [3]. According to the catecholaldehyde hypothesis, DOPAL is central to the degenerative process in Parkinson’s disease [4,5].

Two functional abnormalities tend to build up cytoplasmic DOPAL in residual dopaminergic terminals—decreased vesicular monoamine transporter 2 (VMAT2) activity that is responsible to the vesicular sequestration of cytoplasmic catecholamines [6,7,8] and decreased metabolism of DOPAL by aldehyde dehydrogenase (ALDH) [6,9]. DOPAL, in turn, inhibits the activities of tyrosine hydroxylase [10] and L-aromatic amino acid decarboxylase (LAAAD) [11] and decreases transmembrane DA uptake [5]. A mathematical model of empirical data on catechols derived from cardiac sympathetic innervation in Parkinson’s disease revealed similar dysfunction in catecholamine metabolism, storage, and fate [12]. 

### The Molecular Basis of the Catecholaldehyde Hypothesis

The catecholaldehyde hypothesis is founded on changes in biochemical–cellular processes resulting from a decrease in ALDH and VMAT2 activity and DOPAL accumulation. A better understanding of the array of pathological changes requires a deeper understanding of the molecular basis of these abnormalities. The storage of monoamines in vesicles requires a transporter that will efficiently and quickly transfer them from the cytoplasm against a particularly high concentration and an acidic and electrochemical gradient [13]. Much information about the significance of VMAT2 in Parkinson’s disease was learned from studies conducted on mice that are heterozygous for VMAT2 [14] or VMAT2-deficient mice [15], where a decrease in the activity of the transporter was shown to be strongly related to Parkinson’s disease [16]. 

The other determinant of DOPAL accumulation is a decrease in ALDH activity. A decrease in VMAT2 activity by itself could lead to a shift of dopamine from within the vesicle into the cytoplasm, where an effective metabolism of the cytoplasmic dopamine through MAO to form DOPAL, which is turned immediately and efficiently to DOPAC through ALDH, would at least prevent the accumulation of DOPAL, and thus, its neurotoxicity. With intact ALDH, dopaminergic neurons with reduced vesicular sequestration would have been dysfunctional, but there was no extensive destruction of these neurons as occurs in the disease. In Parkinson’s disease, however, ALDH activity is impaired, and therefore the detoxification capacity of catecholadehydes is impaired, which leads to the neurodegenerative process [17,18]. This is the fundamental rationale of the double-hit concept, which is supported by neurochemical findings in the post-mortems of Parkinson’s disease patients. ALDH dysfunction has been implicated in a number of neurological disorders, including Parkinson’s disease. ALDH dysfunction in Parkinson’s disease may be due to genetic factors and mitochondrial dysfunction [6,18,19,20]. 

Until recently, no animal model had been shown to reproduce the pattern of brain catecholaminergic abnormalities found in Parkinson’s disease—in particular, decreased vesicular sequestration, decreased activities of LAAAD and ALDH, and DOPAL buildup. Recently, we analyzed the striatal neurochemical profile following systemic administration of rotenone to Sprague Dawley rats, a well-established animal model of Parkinson’s disease [21,22,23]. Rotenone caused striatal depletion of dopamine, increased striatal DOPAL, and induced the double hit of decreased vesicular sequestration and decreased ALDH activity [24]. We therefore concluded that the rat rotenone model replicates the changes found in humans and is in line with the neurochemical profile that underlies the catecholaldehyde hypothesis. 

To test the catecholaldehyde hypothesis, it is imperative to demonstrate a beneficial effect of an intervention aimed at lowering DOPAL levels. This was the primary purpose of the present study. We assessed the locomotor effects of combining rotenone with deprenyl, an MAO inhibitor (MAOI) that is known to decrease DOPAL production [25] and is used as an anti-parkinsonian drug [26].

In our mathematical modeling of the phases of the progression of Parkinson’s disease from initiation to its final stages, it seems that the likelihood of disease modifying strategies succeeding depends on the timing [27], with maximal chances to benefit when intervention is initiated early. In this study, we therefore treated animals with deprenyl soon after rotenone administration began. MAO inhibition increases cytoplasmic dopamine levels (because of the vesicular sequestration defect in Parkinson’s disease) which in turn go through spontaneous oxidation to cysDA. In the rat pheochromocytoma cell line (PC12) treated with MAOI and to which antioxidant NAC was added as an adjuvant therapy, there was reduced MAOI-induced increase in cysDA levels and an enhanced beneficial effect of the MAOI treatment [28]. We therefore included in the study an additional intervention—combined treatment with MAOI and NAC to rotenone-treated animals.

In this study, rats received rotenone, rotenone and deprenyl, rotenone and deprenyl and NAC, or sham treatment via subcutaneous reservoir minipumps. Locomotor tests were conducted to verify induction of a parkinsonian movement disorder. Brain tissue of the striatum region was analyzed for levels and ratios of endogenous catechols. From the concept diagram in Figure 1, if there was decreased vesicular sequestration of cytoplasmic catecholamines, then brain tissue DA/DOPAL, DA/DOPAC, and NE/3,4-dihydroxyphenylglycol (DHPG) ratios would be decreased. If there was decreased ALDH activity, the ratio of DOPAC/DOPAL would be decreased.

## 2. Results

Administering rotenone by continuous subcutaneous infusion resulted in gradual weight loss. The group treated with rotenone lost 52.1 ± 21.9 g of weight, representing a 15% decrease from baseline, compared to the control group, which gained 79.3 + 2.8 g during the four weeks of the experiment (*p* = 0.01).

### 2.1. Rotenone Induces Neurochemical and Functional Abnormlities

**Figure 2:**
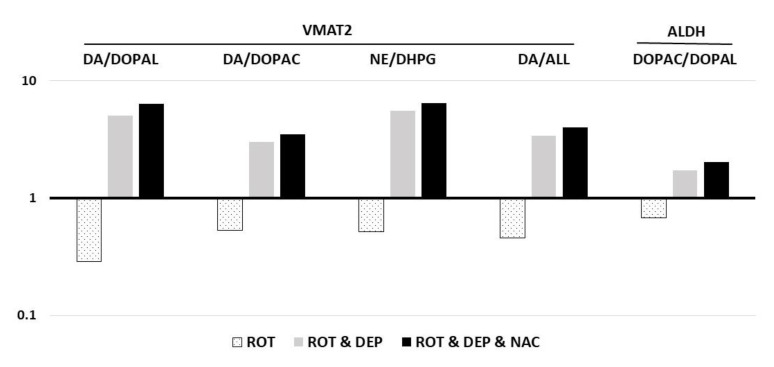
Change (logarithmic scale), compared to vehicle (VEH) in the activity of vesicular monoamine transporter type 2 (VMAT2), as expressed by DA/DOPAL, DA/DOPAC, NE/DHPG, and DA/ALL ratios; and aldehyde dehydrogenase (ALDH), expressed by DOPAC/DOPAL ratio, in rats treated with rotenone (ROT), ROT and deprenyl (DEP), and ROT with DEP and N-acetylcysteine (NAC). Mean values of the intervention group were divided by the mean value of the vehicle in each ratio (Table 2). Note the decrease in the activity of these processes with rotenone and the mitigating effect of the intervention with DEP alone or with NAC. **Abbreviations of catechols:** DHPG: 3,4-dihydroxyphenylglycol; NE: norepinephrine; DOPAL: 3,4-dihydroxyphenylacetaldehyde; DA: dopamine; DOPAC: 3,4-dihydroxyphenylacetic acid.

The effect of rotenone on the neurochemical indices showed a significant decrease in dopamine and an increase in DOPAL and DHPG (Table 1). In addition, the neurochemical profile demonstrates a significant effect of rotenone on the two processes that determine DOAPL levels (Table 2): a decrease in VMAT2 and ALDH activity (Figure 2). 

Parallel to the neurochemical changes, at the end of the study (day 28), rotenone induced motor impairment, with a reduction in the amount of rearing (*p* = 0.019 compared to the control group); and on the open-field test indices, there was reduction in the distance the animal moved (*p* < 0.0001) and their velocity (*p* < 0.001), compared to the control group (Table 3).

### 2.2. Deprrenyl Alone or with NAC Mitigate the Rotenone-Induced Neurochemical and Functional Abnormlities

The addition of deprenyl to rats receiving rotenone reversed the neurochemical pattern induced by rotenone alone, increased dopamine levels, and decreased the aldehyde metabolites DOPAL and DHPG (Table 1). Deprenyl also reversed the negative effect of rotenone on the activity of VMAT2 and ALDH (Table 2, Figure 2).

At the end of the study (day 28), deprenyl added to rotenone-treated rats yielded an increase in the number of rearing (*p* = 0.028), the distance (*p* = 0.048), and the velocity (*p* = 0.036), compared to rotenone without deprenyl (Table 3).

Examining the effect of NAC added to deprenyl treatment in rotenone-treated rats on the neurochemical profile showed a further decrease in DOPAL and a further increase in dopamine (Table 1). This change was not associated with change in the motor indices (Table 3).

## 3. Discussion

Parkinson’s disease is a neurodegenerative disease whose treatment is mainly symptomatic. Understanding mechanisms is a means and a critical step for developing drugs that can modify the progression of the disease [29]. The catecholaldehyde hypothesis centers on accumulation of the autotoxin DOPAL, an obligatory intermediate of intra-neuronal cytoplasmic dopamine. DOPAL accumulation in dopaminergic cells is cytotoxic [30,31]. A “double hit” of decreased ALDH and VMAT2 activity causes the accumulation (Figure 1). Recently, we showed that rotenone given continuously to rats induces the locomotor and neurochemical impairments typical of Parkinson’s disease. To establish a role for DOPAL in inducing the features of the disease, we tested in the rotenone model whether an intervention that decreases DOPAL production improves the parkinsonian phenotype. This demonstration supports the catecholaldehyde hypothesis for the distinct motor phenotype of the disease.

Our results confirmed that rotenone increases DOPAL levels by decreasing VMAT2 and ALDH activities. These findings repeat what was described in more detail in the original studies presenting the rotenone Parkinson’s disease model [21,24]. Administering an MAOI significantly lowered the DOPAL levels, restored VMAT2 and ALDH activity, and improved movement impairment. It is important to note that deprenyl itself has no motor effects in a non-parkinsonian setting [32]. We used deprenyl as a pharmacological means to reduce DOPAL. This was not the purpose for which this molecule was developed, which was to create a means to increase dopamine levels in neurons that are involved in Parkinson’s disease [26]. In our model, this property of the drug was confirmed as expected. We have now shown that deprenyl also reduces DOPAL levels. 

### 3.1. The Centrality of DOPAL in Dopamine Neuronal Toxicity

A growing body of evidence indicates that DOPAL accumulation is toxic, due to oxidative stress, mitochondrial damage, and protein changes (particularly pathological aggregation of alpha-synuclein), all of which are linked to the development and progression of Parkinson’s disease [33,34,35,36,37]. The interaction between DOPAL and alpha-synuclein is complex and includes several mechanisms. DOPAL was found to bind covalently to alpha-synuclein, which leads to the formation of adducts that modify the protein structure and increase the propensity to oligomerize. DOPAL has also been shown to increase the fibrillization of alpha-synuclein, leading to the formation of more toxic and stable aggregates. DOPAL impairs the cellular ability to clear these aggregates; consequently, larger, and more stable aggregates are created. The toxicity of these aggregates derives from their ability to disrupt normal cellular processes, impair synaptic function, and later cause neuronal death. Moreover, DOPAL exerts deleterious effects on other processes such as inducing oxidative stress [38], impairing mitochondrial activity [39], quinonizing other proteins [34], reducing neuronal resilience [40], and collectively accelerating cytotoxic processes leading to the death of dopaminergic nerve cells. A recent comprehensive review details the relationship between alpha-synuclein and deprenyl, the MAOI we chose to reduce DOPAL, and it suggests that MAOIs counteract the deleterious effects on mitochondrial malfunction, oxidative stress, apoptosis, and alpha-synuclein aggregation [41]. Moreover, it has been shown that alpha-synuclein is not as toxic as it is in the presence of DOPAL [42]. 

A variety of cellular processes are adversely affected by DOPAL, but which of these are actually responsible for motor impairment is a challenging question. A comprehensive computational modeling of events in catecholaminergic neurons identified several functional abnormalities in residual dopaminergic nerve terminals, which contribute to depletion of neurotransmitter stores [12], as confirmed in this model [24]. In addition, neural activity might be impaired due to DOPAL-induced damage to the neural projections of dopaminergic neurons [40]. DOPAL also affect the dopamine transporter and thus compromises the normal synaptic activity of dopaminergic neurons [43,44].

### 3.2. Should MAOI for Parkinson’s Disease Be Revisited?

The effect of DOPAL-lowering intervention highlights the promise of mechanism-directed intervention and, in the case of the catecholaldehyde hypothesis, a DOPAL-directed approach. A straightforward treatment is preventing DOPAL production by inhibiting MAO. Disappointingly, the clinical experience with MAOIs did not achieve the expected results. There can be two reasons for this. The first is the fact that the benefit of MAOI-induced increase in dopamine levels in a disease with a vesicular sequestration defect results in the accumulation of dopamine in the cytoplasm rather than the vesicles. The accumulating cytoplasmic dopamine then spontaneously oxidizes to products such as aminochrome and 5-S-cysteinyl DA (cysDA), which are themselves neurotoxic [45,46,47]. Thus, the benefit of lowering DOPAL was partially or even largely mitigated by accumulating these toxic oxidative products. Support for this explanation can be found in a study on PC12 cell culture, where cells were treated to increase DOPAL [28]. The administration of an MAOI did reduce DOPAL levels but at the cost of an increase in cysDA. In that model, the addition of the antioxidant NAC neutralized the spontaneous oxidation of dopamine and prevented the increase in cysDA. In our study, we therefore also tested the effect of the addition of NAC to the treatment of MAOI. It is important to note that not all antioxidants remain active after the first pass through the liver, and not every antioxidant passes the blood–brain barrier sufficiently. Studies examining the effect of antioxidants on patients with Parkinson’s disease have shown mixed results [48,49,50,51]. In our study, we chose NAC due to (1) the experience with this antioxidant in the PC12 model, as noted above; (2) the studies that demonstrated blood–brain barrier penetration, as shown by measuring NAC levels in the spinal fluid after oral administration in patients with Parkinson’s disease [52]; and (3) the fact that NAC had been shown to substantially mitigate DOPAL-induced oligomerization of alpha-synuclein [34]. Our results demonstrated that NAC supplementation increases the DOPAL-lowering effect of MAOI in the rotenone model.

Another possible reason for the failure of MAOIs in clinical trials is related to the timing of the intervention. In those studies, the pharmacological intervention was given to symptomatic patients, most of them several years after diagnosis. Mathematical modeling of the neurodegenerative progression in Parkinson’s disease has identified three phases: homeostasis, dyshomeostasis, and advanced neurodegenerative, when symptoms are evident [27]. Intervention in the advanced phase can improve symptoms but does not affect the course of the disease, since most of the relevant neurons have already been destroyed. This model may explain the failure of MAOI as a disease-modifying strategy. According to this mathematical model, early intervention would substantially delay the onset of symptomatic disease. In our study, we therefore chose to initiate the pharmacological intervention immediately upon the onset of disease induction. This information may open a field of treatment opportunities by influencing the DOPAL level in dopaminergic neurons, either through reducing production or increasing detoxification; treatments targeting the various interactions of DOPAL with alpha-synuclein; and the effects of DOPAL on other cellular proteins and the formation of oxidative stress.

### 3.3. Study’s Limitations and Strengths

The study presents several limitations: We did not test the dose–response and time–response relationships, nor did we test the entirety of rotenone’s motor effects, and only representative tests. We did not examine neuropathology as another outcome of the DOPAL-lowering effect. Concerning the latter, our primary objective in this study was to link neurochemical abnormalities with functional impairment in an animal model of Parkinson’s disease, specifically to associate DOPAL levels with locomotor indices. The study design did not include neuropathological data for two reasons: feasibility and conceptual. Neurochemical analysis of striatal tissue for catechols does not allow the use of formaldehyde, yet neuropathological analysis depends on that. Therefore, we relied on available neuropathological data on rotenone and DOPAL for the specified purpose of this functional–neurochemical study [3,21,22,53]. Regarding the effect of MAOI, based on the large body of evidence about MAOI in Parkinson’s disease, the primary pharmacological effect is increasing neuronal dopamine, thereby improving locomotor function, which may or may not be directly related to observable neuropathological changes. This is consistent with our understanding that efforts should be directed toward correcting functional abnormalities. These limitations are important but do not compromise the findings or their interpretation. As to the study’s strengths, this is the first reported test of the catecholaldehyde hypothesis in an animal model of Parkinson’s disease. We also point to the potential of combination therapy for Parkinson’s disease—MAOI and NAC. Lastly, this report used a model that reflects multiple biochemical lesions similar to what have been found in Parkinson’s disease patients. 

In summary, in the rotenone model, motor impairment typical of Parkinson’s disease is observed parallel to DOPAL increase, secondary to a combined defect in VMAT and ALDH. Lowering DOPAL levels improves the rotenone-induced parkinsonian motor impairment. Reducing DOPAL is achieved by inhibiting the enzyme responsible for its production—MAO. This inhibition is enhanced by adding the central antioxidant NAC. These data pave the way for expanding research on the catecholaldehyde hypothesis and its translation to therapeutics. It is necessary to complete the characterization of the model, especially expand on alpha-synuclein and neural function, and to test neuroprotective strategies by interventions targeting specific processes or combined treatment (such as MAOI with NAC), strategies that can be ascertained clinically through objective biomarkers.

## 4. Material and Methods

The animal research procedures in this study were in accordance with the guidelines of the Chaim Sheba Medical Center and approved by the Animal Care Committee, Sheba Medical Center, Tel Hashomer. The investigators were not blinded as to the treatment conditions.

### 4.1. Animals

Sprague Dawley male rats (350 ± 30 g, 10 weeks old) were obtained from Envigo RMS (Jerusalem, Israel). The rats had at least 3 days to acclimate before surgery (minipump implantation) (BioTest, Kfar-Saba, Israel) and experiments. After the surgery, the rats were housed separately in cages in an animal care facility at 22 °C with a 14 h light (6:00–20:00) and 10 h dark (20:00–6:00) cycle, with free access to food and water. Body weight was measured once a week.

### 4.2. Materials

Dimethyl sulfoxide (DMSO) (Sigma, Rehovot, Israel) and polyethylene glycol (PEG) (CS Chemicals, Haifa, Israel) were mixed in a 1:1 ratio and used as a vehicle to emulsify rotenone. Rotenone was used emulsified in a 6.7 mg/mL DMSO/PEG mixture and injected into an Alzet minipump until the pump was full. For vehicle-treated rats, the 1:1 DMSO/PEG mixture was used alone. Deprenyl (Sigma, Rehovot, Israel) was prepared in a concentration of 1.75 mg/mL in 0.9% sodium chloride for injection.

### 4.3. Study Protocol

For minipump insertion, rats were anesthetized using 3% isoflurane and placed in a small animal stereotaxic frame. An Alzet minipump was implanted subcutaneously at the dorsum of the neck. Rotenone was dissolved in equal volumes of DMSO/PEG vehicle and administered continuously via the minipump at 2 mg/kg/day. In the rat rotenone model [22], the behavioral phenotype is evident by several days of treatment in adult rats. We therefore set the duration of the current study to 28 days. Rats were treated with a dosage of 5 mg/kg/day deprenyl dissolved in normal saline and administered via intraperitoneal injection.

Rats were stratified into 4 groups: (1) vehicle-treated group (*n* = 10); (2) rotenone-treated group (*n* = 8); (3) rotenone and deprenyl-treated group (*n* = 8); (4) rotenone and deprenyl and NAC treated group (*n* = 8). The duration of the study for each rat was 28 days. Group 1 (vehicle treatment) underwent minipump implantation subcutaneously, and the pump contained only the vehicle. Group 2 (rotenone), Group 3 (rotenone+deprenyl) and Group 4 (rotenone+deprenyl+NAC) underwent minipump implantation, and the pump contained rotenone in the vehicle. Groups 3 and 4 also received daily intraperitoneal injections of deprenyl, and group 4 received NAC in drinking water (1 mg/kg/day). The rats were in metabolic cages to ascertain the amount of NAC they drank.

On day 28, the animals were euthanized by 3% isoflurane overdose. Dissections took place immediately after sacrifice. The brain was removed, tissue of the striatum was dissected out on an ice pack. Each sample (approximately 3 mm × 3 mm× 3 mm) was placed in a plastic cryotube, frozen immediately in liquid nitrogen, and stored at −80 °C until assayed.

### 4.4. Locomotor Tests

The rearing and open field tests are a widely used tool in animal models for stroke and Parkinson’s disease [54,55]. Locomotor tests were carried out at bassline and on day 28. We repeated the tests we described previously [24]. Briefly, for the open field test, rats were placed in an apparatus (measurements—100 cm × 100 cm × 30 cm for length, width and height) and were allowed free exploration while being videotaped for 5 min. The recordings were analyzed for distance and average velocity during this period. For rearing, rats were placed in a rectangular plastic apparatus (measurements—30 cm × 34 cm × 16 cm for length, width, and height) and videotaped for 2 min to record spontaneous rearing behavior. Total rearing number was the number of times the animal was leaning on its hind legs and dethatching its forelegs. This parameter allowed evaluation of the ability of the rat to perform activities that require physical strength, coordination, and balance.

### 4.5. Neurochemical Assays

Tissue samples were transferred in dry ice to the catecholamine resource facility, thawed at room temperature and homogenized in dilute 20% 0.2 M phosphoric acid, 80% 0.2 M acetic acid in a fume hood. Aliquots of the supernatant were assayed for catechol contents by batch alumina extraction followed by liquid chromatography with series electrochemical detection, as described previously [56]. NE, DOPAC, DHPG, DOPAL, DOPET, DOPA, and dopamine were assayed simultaneously. Tissue concentrations were expressed in pmol/mg wet weight.

### 4.6. Statistics

Data analysis was performed with IBM SPSS 23, using analyses of variance followed by Tukey’s post-hoc test. Independent-means *t*-tests were used to compare the results of biochemical analyses between the vehicle- and rotenone-treated groups and between the rotenone alone vs. rotenone + deprenyl groups and rotenone + deprenyl + NAC vs. rotenone+deprenyl groups. Locomotor data were generated using a video tracking software (EthoVision v.15, Wageningen, The Netherlands). The neurochemical assay personnel were blinded to the treatment conditions until the results were tabulated. Mean values ± SEM are shown in Table 1. Statistical significance was defined by a *p* value < 0.05.

## Figures and Tables

**Table 1:** Brain tissue catechols (means ± SEM) in rats treated with vehicle (VEH), rotenone (ROT), or rotenone and deprenyl (ROT+DEP), or rotenone and deprenyl and N-acetylcysteine (ROT+DEP+NAC). Below the table—statistically significant differences between groups in each parameter.

Catechol	VEH	ROT	ROT+DEP	ROT+DEP+NAC
DHPG	50.37 ± 3.96 ^abc^	96.17 ± 5.06 ^ade^	27.71 ± 4.87 ^bd^	29.46 ± 2.37 ^ce^
NE	112.90 ± 11.38 ^bc^	112.03 ± 11.4 ^de^	284.45 ± 27.81 ^bdf^	422.02 ± 50.54 ^cef^
DOPA	44.28 ± 3.51 ^abc^	65.53 ± 4.33 ^ade^	32.34 ± 1.41 ^bd^	29.66 ± 1.44 ^ce^
DOPAL	685.94 ± 72.6 ^abc^	1152.1 ± 97.38 ^ade^	429.46 ± 96.88 ^b*df^	192.6 ± 37.93 ^cef^
DA	2665.91 ± 203.7 ^abc^	1417.14 ± 101.42 ^ade^	6304.3 ± 482.63 ^bdf^	4621.73 ± 1078.77 ^cef^
DOPAC	957.93 ± 88.81 ^c^	1029.42 ± 133.55 ^e^	754.8 ± 88.80	516.14 ± 300.14 ^ce^
DOPET	110.67 ± 10.86 ^bc^	125.68 ± 26.76 ^de^	52.09 ± 7.55 ^bdf^	23.28 ± 17.43 ^cef^
CysDA	25.05 ± 2.33 ^abc^	13.75 ± 1.61 ^ad^	48.78 ± 13.04 ^bd^	53.51 ± 14.28 ^c^

**Abbreviations:** DHPG: 3,4-dihydroxyphenylglycol; NE: norepinephrine; DOPA: 3,4-dihydroxyphenylalanine; DOPAL: 3,4-dihydroxyphenylacetaldehyde; DA: dopamine; DOPAC: 3,4-dihydroxyphenylacetic acid; CysDA: cysteinyl-dopamine. **Statistics:** ^a^: *p* < 0.05 for VEH vs. ROT; ^b^: *p* < 0.05 for VEH vs. ROT+DEP; ^c^: *p* < 0.05 for VEH vs. ROT+DEP+NAC; ^d^: *p* < 0.05 for ROT vs. ROT+DEP; ^e^: *p* < 0.05 for ROT vs. ROT+DEP+NAC; ^f^: *p* < 0.05 for ROT+DEP vs. ROT+DEP+NAC. * Not significant in analysis of variance with multiple comparisons. Number of animals treated with vehicle—10, and all other intervention groups—8 animals per group.

**Table 2:** Catechol ratio and the process reflected (means ± SEM) in rats treated with vehicle (VEH), rotenone (ROT), rotenone and deprenyl (ROT+DEP), or rotenone and deprenyl and N-acetylcysteine (ROT+DEP+NAC). Below the table—statistically significant differences between groups in each parameter.

Catechol Ratio	Process	VEH	ROT	ROT+DEP	ROT+DEP+NAC
DA/DOPAL	VMAT2	4.42 ± 0.64 ^abc^	1.27 ± 0.13 ^ade^	22.27 ± 5.7 ^bd^	28.22 ± 4.14 ^ce^
DA/DOPAC	VMAT2	3.01 ± 0.34 ^abc^	1.6 ± 0.3 ^ade^	9.11 ± 1.3 ^bd^	10.5 ± 1.11 ^ce^
NE/DHPG	VMAT2	2.28 ± 0.20 ^abc^	1.18 ± 0.12 ^ade^	12.68 ± 2.46 ^bd^	14.61 ± 1.43 ^ce^
DA/ALL	VMAT2	1.72 ± 0.21 ^ac^	0.79 ± 0.18 ^ade^	5.89 ± 1.01 ^d^	6.92 ± 0.54 ^ce^
DOPAC/DOPAL	ALDH	1.43 ± 0.07 ^abc^	0.98 ± 0.14 ^ade^	2.48 ± 0.49 ^bd^	2.89 ± 0.45 ^ce^

**Abbreviations:** DHPG: 3,4-dihydroxyphenylglycol; NE: norepinephrine; DOPA: 3,4-dihydroxyphenylalanine; DOPAL: 3,4-dihydroxyphenylacetaldehyde; DA: dopamine; DOPAC: 3,4-dihydroxyphenylacetic acid; VMAT: vesicular monoamine transporter type 2; ALDH: aldehyde dehydrogenase. **Statistics:** ^a^: *p* < 0.05 for VEH vs. ROT; ^b^: *p* < 0.05 for VEH vs. ROT+DEP; ^c^: *p* < 0.05 for VEH vs. ROT+DEP+NAC; ^d^: *p* < 0.05 for ROT vs. ROT+DEP; ^e^: *p* < 0.05 for ROT vs. ROT+DEP+NAC.

**Table 3:** Locomotor parameters and the day that they were recorded (means ± SEM) in rats treated with vehicle (VEH), rotenone (ROT), rotenone and deprenyl (ROT+DEP), or rotenone and deprenyl and N-acetylcysteine (ROT+DEP+NAC). Below the table—statistically significant differences between groups in each parameter.

Motor Parameter	Day	VEH	ROT	ROT+DEP	ROT+DEP+NAC
Rearing (counts)	BL	6.50 ± 0.89	4.18 ± 0.69	6.00 ± 1.20	6.00 ± 0.73
	End	3.8 ± 0.53 ^a^	2.00 ± 0.38 ^ade^	3.57 ± 0.48 ^d^	3.57 ± 0.30 ^e^
Distance (cm)	BL	2962.3 ± 240.8	2831.4 ± 250.0	1328.0 ± 524.3	2325.2 ± 346.6
	END	2291.1 ± 201.9 ^abc^	972.8 ± 132.7 ^ade^	1540.5 ± 259.84 ^bd^	1765.9 ± 67.9 ^ce^
Velocity (cm/min)	BL	9.87 ± 0.80	9.44 ± 0.83	4.43 ± 1.75	8.31 ± 0.89
	END	7.64 ± 0.68 ^abc^	3.23 ± 0.32 ^ade^	5.02 ± 0.85 ^bd^	5.39 ± 0.39 ^ce^

Abbreviations: BL: baseline. **Statistics:** ^a^: *p* < 0.05 for VEH vs. ROT; ^b^: *p* < 0.05 for VEH vs. ROT+DEP; ^c^: *p* < 0.05 for VEH vs. ROT+DEP+NAC; ^d^: *p* < 0.05 for ROT vs. ROT+DEP; ^e^: *p* < 0.05 for ROT vs. ROT+DEP+NAC.

## Data Availability

The data that support the reported results are available on reasonable request from the corresponding author.

## Abbreviations

AS: alpha-synuclein; ALDH: aldehyde dehydrogenase; AR: aldehyde/aldose reductase; CysDA: cysteinyl dopamine; DOPA: 3,4-dihydroxyphenylalanine; DA: dopamine; DAT: cell membrane DA transporter; DBH: dopamine-beta-hydroxylase; DHPG: 3,4-dihydroxyphenylglycol; DOPAL: 3,4-dihydroxyphenylacetaldehyde; DOPAC: 3,4-dihydroxyphenylacetic acid; DOPEGAL: 3,4-dihydroxyphenylglycolaldehyde; MAO: monoamine oxidase; NE: norepinephrine; NET: cell membrane norepinephrine transporter; TH: tyrosine hydroxylase.

## References

[1] Goldstein D.S., Sullivan P., Holmes C., Mash D.C., Kopin I.J., Sharabi Y. (2017). Determinants of denervation-independent depletion of putamen dopamine in Parkinson’s disease and multiple system atrophy. Park. Relat. Disord..

[2] Goldstein D.S., Holmes C., Sullivan P., Mash D.C., Sidransky E., Stefani A., Kopin I.J., Sharabi Y. (2015). Deficient vesicular storage: A common theme in catecholaminergic neurodegeneration. Park. Relat. Disord..

[3] Panneton W.M., Kumar V.B., Gan Q., Burke W.J., Galvin J.E. (2010). The neurotoxicity of DOPAL: Behavioral and stereological evidence for its role in Parkinson disease pathogenesis. PLoS ONE.

[4] Burke W.J., Li S.W., Williams E.A., Nonneman R., Zahm D.S. (2003). 3,4-Dihydroxyphenylacetaldehyde is the toxic dopamine metabolite in vivo: Implications for Parkinson’s disease pathogenesis. Brain Res..

[5] Mattammal M.B., Haring J.H., Chung H.D., Raghu G., Strong R. (1995). An endogenous dopaminergic neurotoxin: Implication for Parkinson’s disease. Neurodegeneration.

[6] Goldstein D.S., Sullivan P., Holmes C., Miller G.W., Alter S., Strong R., Mash D.C., Kopin I.J., Sharabi Y. (2013). Determinants of buildup of the toxic dopamine metabolite DOPAL in Parkinson’s disease. J. Neurochem..

[7] Miller G.W., Erickson J.D., Perez J.T., Penland S.N., Mash D.C., Rye D.B., Levey A.I. (1999). Immunochemical analysis of vesicular monoamine transporter (VMAT2) protein in Parkinson’s disease. Exp. Neurol..

[8] Okamura N., Villemagne V.L., Drago J., Pejoska S., Dhamija R.K., Mulligan R.S., Ellis J.R., Ackermann U., O’Keefe G., Jones G. (2010). In vivo measurement of vesicular monoamine transporter type 2 density in Parkinson disease with (18)F-AV-133. J. Nucl. Med. Off. Publ. Soc. Nucl. Med..

[9] Fitzmaurice A.G., Rhodes S.L., Lulla A., Murphy N.P., Lam H.A., O’Donnell K.C., Barnhill L., Casida J.E., Cockburn M., Sagasti A. (2013). Aldehyde dehydrogenase inhibition as a pathogenic mechanism in Parkinson disease. Proc. Natl. Acad. Sci. USA.

[10] Vermeer L.M., Florang V.R., Doorn J.A. (2012). Catechol and aldehyde moieties of 3,4-dihydroxyphenylacetaldehyde contribute to tyrosine hydroxylase inhibition and neurotoxicity. Brain Res..

[11] Jinsmaa Y., Sharabi Y., Sullivan P., Isonaka R., Goldstein D.S. (2018). 3,4-Dihydroxyphenylacetaldehyde-Induced Protein Modifications and Their Mitigation by N-Acetylcysteine. J. Pharmacol. Exp. Ther..

[12] Goldstein D.S., Pekker M.J., Eisenhofer G., Sharabi Y. (2019). Computational modeling reveals multiple abnormalities of myocardial noradrenergic function in Lewy body diseases. JCI Insight.

[13] Wimalasena K. (2011). Vesicular monoamine transporters: Structure-function, pharmacology, and medicinal chemistry. Med. Res. Rev..

[14] Takahashi N., Miner L.L., Sora I., Ujike H., Revay R.S., Kostic V., Jackson-Lewis V., Przedborski S., Uhl G.R. (1997). VMAT2 knockout mice: Heterozygotes display reduced amphetamine-conditioned reward, enhanced amphetamine locomotion, and enhanced MPTP toxicity. Proc. Natl. Acad. Sci. USA.

[15] Taylor T.N., Caudle W.M., Miller G.W. (2011). VMAT2-Deficient Mice Display Nigral and Extranigral Pathology and Motor and Nonmotor Symptoms of Parkinson’s Disease. Park. Dis..

[16] Alter S.P., Lenzi G.M., Bernstein A.I., Miller G.W. (2013). Vesicular integrity in Parkinson’s disease. Curr. Neurol. Neurosci. Rep..

[17] Deza-Ponzio R., Herrera M.L., Bellini M.J., Virgolini M.B., Herenu C.B. (2018). Aldehyde dehydrogenase 2 in the spotlight: The link between mitochondria and neurodegeneration. Neurotoxicology.

[18] Grunblatt E., Riederer P. (2016). Aldehyde dehydrogenase (ALDH) in Alzheimer’s and Parkinson’s disease. J. Neural Transm..

[19] Chen C.H., Joshi A.U., Mochly-Rosen D. (2016). The Role of Mitochondrial Aldehyde Dehydrogenase 2 (ALDH2) in Neuropathology and Neurodegeneration. Acta Neurol. Taiwanica.

[20] Wey M.C., Fernandez E., Martinez P.A., Sullivan P., Goldstein D.S., Strong R. (2012). Neurodegeneration and motor dysfunction in mice lacking cytosolic and mitochondrial aldehyde dehydrogenases: Implications for Parkinson’s disease. PLoS ONE.

[21] Betarbet R., Sherer T.B., MacKenzie G., Garcia-Osuna M., Panov A.V., Greenamyre J.T. (2000). Chronic systemic pesticide exposure reproduces features of Parkinson’s disease. Nat. Neurosci..

[22] Cannon J.R., Tapias V., Na H.M., Honick A.S., Drolet R.E., Greenamyre J.T. (2009). A highly reproducible rotenone model of Parkinson’s disease. Neurobiol. Dis..

[23] Zhang Z.N., Zhang J.S., Xiang J., Yu Z.H., Zhang W., Cai M., Li X.T., Wu T., Li W.W., Cai D.F. (2017). Subcutaneous rotenone rat model of Parkinson’s disease: Dose exploration study. Brain Res..

[24] Landau R., Halperin R., Sullivan P., Zibly Z., Leibowitz A., Goldstein D.S., Sharabi Y. (2022). The rat rotenone model reproduces the abnormal pattern of central catecholamine metabolism found in Parkinson’s disease. Dis. Models Mech..

[25] Goldstein D.S., Jinsmaa Y., Sullivan P., Holmes C., Kopin I.J., Sharabi Y. (2016). Comparison of Monoamine Oxidase Inhibitors in Decreasing Production of the Autotoxic Dopamine Metabolite 3,4-Dihydroxyphenylacetaldehyde in PC12 Cells. J. Pharmacol. Exp. Ther..

[26] Riederer P., Youdim M.B. (1986). Monoamine oxidase activity and monoamine metabolism in brains of parkinsonian patients treated with l-deprenyl. J. Neurochem..

[27] Goldstein D.S., Pekker M.J., Sullivan P., Isonaka R., Sharabi Y. (2022). Modeling the Progression of Cardiac Catecholamine Deficiency in Lewy Body Diseases. J. Am. Heart Assoc..

[28] Goldstein D.S., Jinsmaa Y., Sullivan P., Sharabi Y. (2017). N-Acetylcysteine Prevents the Increase in Spontaneous Oxidation of Dopamine During Monoamine Oxidase Inhibition in PC12 Cells. Neurochem. Res..

[29] Anton S.D. (2014). Can non-nutritive sweeteners enhance outcomes of weight loss interventions?. Obesity.

[30] Goldstein D.S. (2021). The Catecholaldehyde Hypothesis for the Pathogenesis of Catecholaminergic Neurodegeneration: What We Know and What We Do Not Know. Int. J. Mol. Sci..

[31] Masato A., Plotegher N., Boassa D., Bubacco L. (2019). Impaired dopamine metabolism in Parkinson’s disease pathogenesis. Mol. Neurodegener..

[32] Wahdan S.A., Tadros M.G., Khalifa A.E. (2017). Antioxidant and antiapoptotic actions of selegiline protect against 3-NP-induced neurotoxicity in rats. Naunyn-Schmiedeberg’s Arch. Pharmacol..

[33] Carmo-Goncalves P., Coelho-Cerqueira E., de Araujo Lima V., Follmer C. (2023). Alpha-synuclein in Parkinson’s disease: A villain or tragic hero? A critical view of the formation of alpha-synuclein aggregates induced by dopamine metabolites and viral infection. Expert Rev. Neurother..

[34] Jinsmaa Y., Isonaka R., Sharabi Y., Goldstein D.S. (2020). 3,4-Dihydroxyphenylacetaldehyde Is More Efficient than Dopamine in Oligomerizing and Quinonizing alpha-Synuclein. J. Pharmacol. Exp. Ther..

[35] Kumar V.B., Hsu F.F., Lakshmi V.M., Gillespie K.N., Burke W.J. (2019). Aldehyde adducts inhibit 3,4-dihydroxyphenylacetaldehyde-induced alpha-synuclein aggregation and toxicity: Implication for Parkinson neuroprotective therapy. Eur. J. Pharmacol..

[36] Lima V.A., do Nascimento L.A., Eliezer D., Follmer C. (2019). Role of Parkinson’s Disease-Linked Mutations and N-Terminal Acetylation on the Oligomerization of alpha-Synuclein Induced by 3,4-Dihydroxyphenylacetaldehyde. ACS Chem. Neurosci..

[37] Sarafian T.A., Yacoub A., Kunz A., Aranki B., Serobyan G., Cohn W., Whitelegge J.P., Watson J.B. (2019). Enhanced mitochondrial inhibition by 3,4-dihydroxyphenyl-acetaldehyde (DOPAL)-oligomerized alpha-synuclein. J. Neurosci. Res..

[38] Li S.W., Lin T.S., Minteer S., Burke W.J. (2001). 3,4-Dihydroxyphenylacetaldehyde and hydrogen peroxide generate a hydroxyl radical: Possible role in Parkinson’s disease pathogenesis. Mol. Brain Res..

[39] Kristal B.S., Conway A.D., Brown A.M., Jain J.C., Ulluci P.A., Li S.W., Burke W.J. (2001). Selective dopaminergic vulnerability: 3,4-dihydroxyphenylacetaldehyde targets mitochondria. Free Radic. Biol. Med..

[40] Masato A., Plotegher N., Terrin F., Sandre M., Faustini G., Thor A., Adams S., Berti G., Cogo S., De Lazzari F. (2023). DOPAL initiates alphaSynuclein-dependent impaired proteostasis and degeneration of neuronal projections in Parkinson’s disease. NPJ Park. Dis..

[41] Naoi M., Maruyama W., Shamoto-Nagai M. (2020). Rasagiline and selegiline modulate mitochondrial homeostasis, intervene apoptosis system and mitigate alpha-synuclein cytotoxicity in disease-modifying therapy for Parkinson’s disease. J. Neural Transm..

[42] Martinez P.A., Martinez V.E., Rani S., Murrell M., Javors M., Gelfond J., Doorn J.A., Fernandez E., Strong R. (2023). Impaired aldehyde detoxification exacerbates motor deficits in an alpha-synuclein mouse model of Parkinson’s disease. Brain Behav..

[43] Longo F., Mercatelli D., Novello S., Arcuri L., Brugnoli A., Vincenzi F., Russo I., Berti G., Mabrouk O.S., Kennedy R.T. (2017). Age-dependent dopamine transporter dysfunction and Serine129 phospho-alpha-synuclein overload in G2019S LRRK2 mice. Acta Neuropathol. Commun..

[44] Stednitz S.J., Freshner B., Shelton S., Shen T., Black D., Gahtan E. (2015). Selective toxicity of L-DOPA to dopamine transporter-expressing neurons and locomotor behavior in zebrafish larvae. Neurotoxicol. Teratol..

[45] Badillo-Ramirez I., Saniger J.M., Rivas-Arancibia S. (2019). 5-S-cysteinyl-dopamine, a neurotoxic endogenous metabolite of dopamine: Implications for Parkinson’s disease. Neurochem. Int..

[46] Herrera A., Munoz P., Steinbusch HW M., Segura-Aguilar J. (2017). Are Dopamine Oxidation Metabolites Involved in the Loss of Dopaminergic Neurons in the Nigrostriatal System in Parkinson’s Disease?. ACS Chem. Neurosci..

[47] Segura-Aguilar J., Paris I., Munoz P., Ferrari E., Zecca L., Zucca F.A. (2014). Protective and toxic roles of dopamine in Parkinson’s disease. J. Neurochem..

[48] Deus C.M., Teixeira J., Raimundo N., Tucci P., Borges F., Saso L., Oliveira P.J. (2022). Modulation of cellular redox environment as a novel therapeutic strategy for Parkinson’s disease. Eur. J. Clin. Investig..

[49] Monti D.A., Zabrecky G., Kremens D., Liang T.W., Wintering N.A., Bazzan A.J., Zhong L., Bowens B.K., Chervoneva I., Intenzo C. (2019). N-Acetyl Cysteine Is Associated with Dopaminergic Improvement in Parkinson’s Disease. Clin. Pharmacol. Ther..

[50] Beal M.F., Oakes D., Shoulson I., Henchcliffe C., Galpern W.R., Haas R., Juncos J.L., Nutt J.G., Voss T.S., Parkinson Study Group QE3 Investigators (2014). A randomized clinical trial of high-dosage coenzyme Q10 in early Parkinson disease: No evidence of benefit. JAMA Neurol..

[51] Prasuhn J., Bruggemann N., Hessler N., Berg D., Gasser T., Brockmann K., Olbrich D., Ziegler A., Konig I.R., Klein C. (2019). An omics-based strategy using coenzyme Q10 in patients with Parkinson’s disease: Concept evaluation in a double-blind randomized placebo-controlled parallel group trial. Neurol. Res. Pract..

[52] Katz M., Won S.J., Park Y., Orr A., Jones D.P., Swanson R.A., Glass G.A. (2015). Cerebrospinal fluid concentrations of N-acetylcysteine after oral administration in Parkinson’s disease. Park. Relat. Disord..

[53] Miyazaki I., Isooka N., Imafuku F., Sun J., Kikuoka R., Furukawa C., Asanuma M. (2020). Chronic Systemic Exposure to Low-Dose Rotenone Induced Central and Peripheral Neuropathology and Motor Deficits in Mice: Reproducible Animal Model of Parkinson’s Disease. Int. J. Mol. Sci..

[54] Hua Y., Schallert T., Keep R.F., Wu J., Hoff J.T., Xi G. (2002). Behavioral tests after intracerebral hemorrhage in the rat. Stroke.

[55] Schaar K.L., Brenneman M.M., Savitz S.I. (2010). Functional assessments in the rodent stroke model. Exp. Transl. Stroke Med..

[56] Holmes C., Eisenhofer G., Goldstein D.S. (1994). Improved assay for plasma dihydroxyphenylacetic acid and other catechols using high-performance liquid chromatography with electrochemical detection. J. Chromatogr. B Biomed. Appl..

